# Targeting CSC-immune cell crosstalk to overcome chemoresistance and enhance immunotherapy efficacy

**DOI:** 10.3389/fimmu.2025.1620807

**Published:** 2025-07-23

**Authors:** Guanxiao Yu, Jianbao Gong

**Affiliations:** Qingdao Hospital, University of Health and Rehabilitation Sciences, Qingdao Municipal Hospital, Qingdao, China

**Keywords:** cancer stem cells (CSCs), tumor immune microenvironment (TIME), immune evasion, therapy resistance, CSC-immune crosstalk

## Abstract

Cancer stem cells (CSCs) are a subpopulation of tumor cells that play crucial roles in driving tumor recurrence, metastasis, and resistance to therapies, including chemotherapy and immunotherapy. Growing evidence suggests that interactions between CSCs and immune cells, particularly tumor-associated macrophages, myeloid-derived suppressor cells, and regulatory T cells, create a supportive tumor microenvironment conducive to immune evasion and chemoresistance. Understanding these intricate crosstalk mechanisms, mediated via cytokines, exosomes, and metabolic intermediates, is crucial for the development of effective therapeutic strategies. Here, we comprehensively review recent progress on CSC-immune cell crosstalk, highlighting key signaling pathways and molecular targets. Furthermore, we discuss promising clinical strategies combining conventional therapies with interventions targeting CSC-immune interactions, aiming to enhance immunotherapy efficacy and overcome therapeutic resistance in cancer patients.

## 1 Introduction

CSCs are specialized tumor cells possessing characteristics of self-renewal, differentiation, and tumor initiation, significantly contributing to metastasis, tumor relapse, and therapeutic resistance (1–4). Despite major advances in cancer therapies, including immunotherapy, CSCs remain a critical barrier due to their inherent resistance mechanisms and their ability to evade immune surveillance (5–7). Emerging studies have demonstrated profound interactions between CSCs and various immune cells in the tumor microenvironment (TME), creating a permissive niche that facilitates tumor progression and resistance to treatments (8–10).

**Figure 1:**
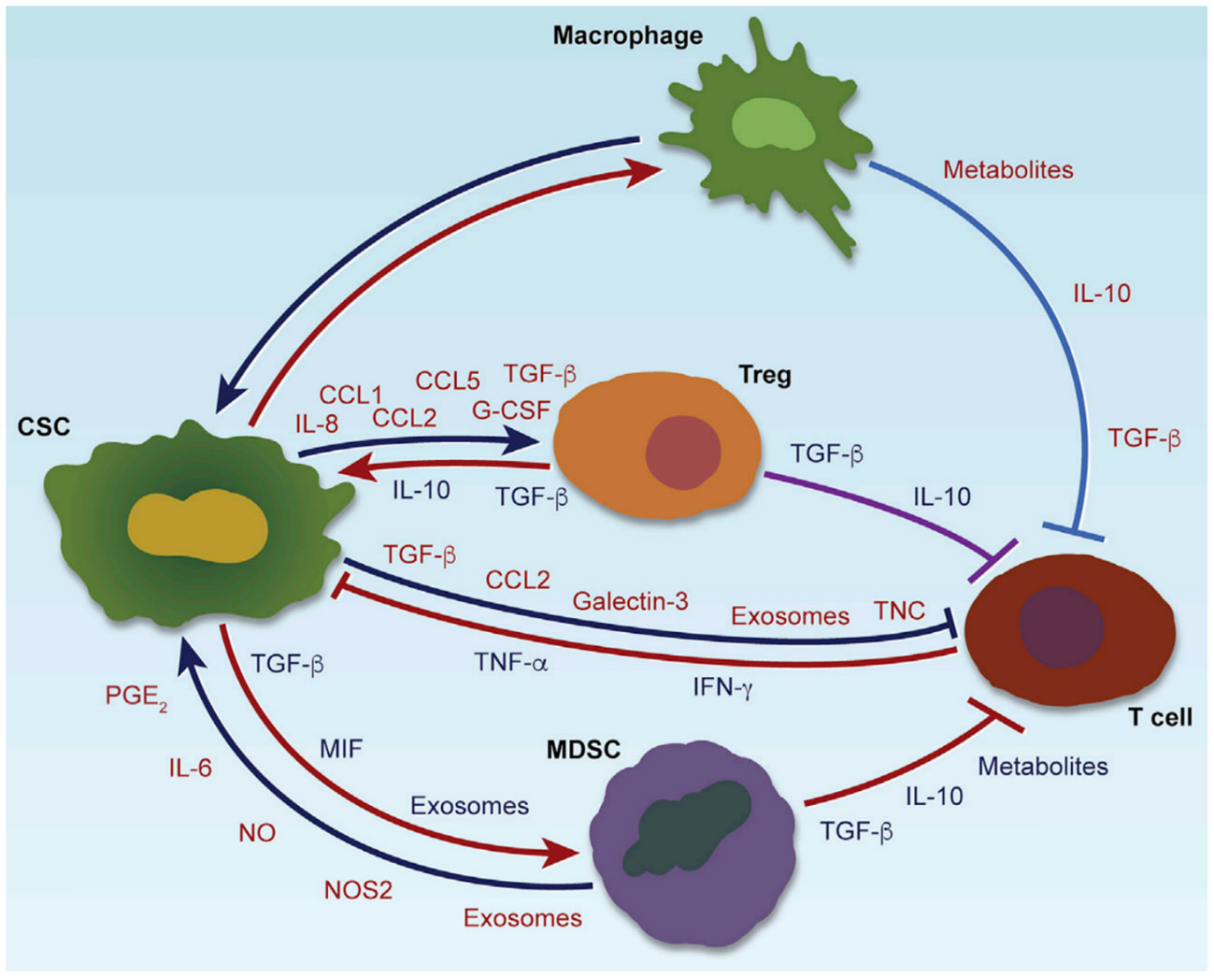
Crosstalk between CSCs and immune cells in the tumor microenvironment.

CSCs profoundly influence the tumor immunity through various immunomodulatory mechanisms. Through the secretion of soluble factors, including immunosuppressive cytokines (such as TGF-β, IL-10) (11, 12), chemokines (such as CCL2, CCL5) (13, 14), and exosomes carrying bioactive molecules (15), CSCs actively recruit and reprogram tumor-associated macrophages (TAMs), myeloid-derived suppressor cells (MDSCs), and regulatory T cells (Tregs) toward immunosuppressive phenotypes (16). These immune cells, once engaged, reciprocally contribute to the maintenance of CSC stemness by providing supportive signals, facilitating immune evasion, promoting epithelial-mesenchymal transition (EMT), and enhancing resistance to chemotherapy and immunotherapy (17, 18). In addition to soluble mediators, metabolic reprogramming within the CSC niche—such as lactate accumulation and adenosine production—further reinforces immune suppression and sustains CSC viability (19). Exosome-mediated communication serves as an additional layer of complexity, allowing CSCs to deliver regulatory RNAs and proteins that modify immune cell behavior at a distance (20). This dynamic and reciprocal crosstalk creates a permissive environment that protects CSCs from immune-mediated elimination and conventional treatments, fueling continuous tumor progression and metastasis. A comprehensive overview of these multifaceted interactions is illustrated in **Figure 1**, and representative therapeutic strategies targeting these interactions are summarized in **Table 1**, highlighting the central role of CSC-driven immune modulation in shaping the tumor microenvironment (7).

**Table 1:** Therapeutic strategies targeting CSC-immune crosstalk and their clinical status.

Strategy	Target	Mechanism	Clinical Status	Representative Agents/Trials
TAM-targeted therapies	CSF-1R, CCL2	Reprogram TAMs to anti-tumor phenotype; reduce CSC niche support	Ongoing (Phase I/II)	Pexidartinib (NCT02777710), Emactuzumab
MDSC-targeted strategies	CXCR1/2, STAT3	Block MDSC recruitment and function; restore T cell activity	Ongoing (Phase I)	SX-682 + Pembrolizumab (NCT03161431)
Treg depletion	CCR4, CD25 (IL-2Rα)	Deplete Tregs to relieve immune suppression	Phase II	Mogamulizumab (NCT02946671), Basiliximab
CSC-specific immunotherapies	CD133, EpCAM, ALDH	Target CSC antigens for immune-mediated killing	Ongoing (Phase I)	CD133-CAR-T cells (NCT03423992, NCT02541370)
CSC signaling pathway inhibitors	Wnt, Notch, Hedgehog pathways	Inhibit CSC self-renewal and reduce immunosuppressive cytokines	Ongoing (Phase I)	LGK974 (Wnt inhibitor) + anti-PD-1 (NCT01351103)
Metabolic modulation	Glutaminase, fatty acid metabolism	Disrupt CSC and immune cell metabolic crosstalk	Ongoing (Phase I/II)	CB-839 (NCT02771626), CPI-613 (NCT03399396)
Epigenetic therapy combinations	DNMT, HDAC	Reverse CSC-driven immune suppression and resistance	Ongoing (Phase I)	Guadecitabine + Atezolizumab (NCT03250273)

The interplay between CSCs and immune cells is orchestrated by several critical signaling cascades including Wnt, Notch, Hedgehog, and PI3K/Akt/mTOR pathways (21). Such pathways have become pivotal therapeutic targets for intervention to disrupt the CSC niche and restore antitumor immunity. Recent translational research and clinical trials emphasize combination therapies that integrate CSC-targeted agents with immunotherapies, showing promising results in overcoming resistance and improving patient outcomes (22, 23). Moreover, CSCs have evolved mechanisms to specifically evade CD8^+^ cytotoxic T cell–mediated recognition and killing, further complicating immunotherapeutic interventions (16, 24). In this mini-review, we focus on the mechanisms of CSC-immune cell interactions and their implications in chemoresistance and immunotherapy efficacy. We systematically outline key molecular pathways mediating this interaction and discuss ongoing efforts to translate these insights into novel, effective clinical strategies. While we focus on CSC-specific mechanisms, we acknowledge that some referenced studies and trials examine broader tumor or immune processes without explicitly isolating CSCs. These findings are interpreted within the context of CSC-related biology and immune modulation (25, 26). By comprehensively understanding these regulatory networks, we aim to pave the way toward more robust therapeutic interventions, effectively targeting CSC-associated immune evasion and therapeutic resistance.

CSCs interact with immune cells through cytokines, chemokines, exosomes, and metabolic factors, shaping an immunosuppressive tumor microenvironment. CSCs recruit and polarize TAMs, MDSCs, and Tregs, which in turn enhance CSC stemness, immune evasion, and therapy resistance. Key pathways involved include Wnt, Notch, Hedgehog, and STAT3 (Color coding in the figure indicates the functional roles of secreted molecules: red for immunosuppression, blue for immune cell recruitment, green for maintenance of stemness or induction of EMT, and orange for promoting therapeutic resistance).

## 2 Immune cell regulation of CSC stemness and chemoresistance

### 2.1 Tumor-associated macrophages

TAMs are prominent immune cells in the TME that significantly influence CSC properties (27–29). They promote CSC stemness through the secretion of growth factors and cytokines such as IL-6, IL-10, and TGF-β. These factors activate crucial signaling pathways including STAT3 and NF-κB within CSCs, thus enhancing CSC self-renewal, survival, and resistance to chemotherapy (30, 31). Additionally, TAMs release pro-inflammatory cytokines (such as TNF-α) and chemokines (CCL2, CXCL8), which promote the expansion and maintenance of CSC populations within the tumor microenvironment (32, 33). Hence, targeting TAM-derived signaling could effectively disrupt CSC niches and reduce therapeutic resistance.

### 2.2 Myeloid-derived suppressor cells

MDSCs are potent immunosuppressive cells that contribute to CSC survival by shaping an immunosuppressive tumor environment. They secrete multiple effector molecules with distinct functions: nitric oxide (NO), reactive oxygen species (ROS), and arginase-1 primarily inhibit antitumor T-cell responses by suppressing T-cell activation and proliferation (34, 35). Meanwhile, immunosuppressive cytokines such as interleukin-10 (IL-10) and transforming growth factor-beta (TGF-β) enhance CSC stemness and chemoresistance by activating key signaling pathways, including NF-κB, PI3K/Akt, and STAT3. This dual mechanism reinforces both immune evasion and CSC-driven tumor progression (12, 36). Additionally, MDSC-derived exosomes contain microRNAs and proteins that reinforce CSC traits. For example, microRNA-21 and microRNA-210, commonly enriched in MDSC exosomes, have been shown to enhance CSC self-renewal and chemoresistance by modulating signaling pathways such as STAT3 and HIF-1α (37, 38). These findings highlight the critical role of MDSCs and their exosomal cargo in maintaining CSC-associated phenotypes (39).

### 2.3 Regulatory T cells

Tregs further promote CSC-mediated tumor progression and therapy resistance (40, 41). Tregs suppress antitumor immune responses through cytokines (TGF-β, IL-10) and cell-contact-dependent mechanisms, facilitating immune escape and indirectly supporting CSC survival and expansion. Moreover, CSC-secreted chemokines (such as CCL1, CCL5) specifically attract Tregs, creating a self-amplifying immunosuppressive loop (42, 43). Blocking the recruitment or suppressive functions of Tregs thus represents a valuable strategy to disrupt CSC immune privilege and enhance therapeutic efficacy.

## 3 Signaling pathways mediating CSC-immune crosstalk

### 3.1 Cytokine and chemokine signaling networks

Cytokines and chemokines form a complex regulatory network mediating bidirectional communication between CSCs and immune cells. CSC-derived cytokines and chemokines extensively reshape immune cell infiltration and polarization within tumors, directly promoting immune evasion and therapeutic resistance (7, 44, 45). For example, TGF−β secreted by CSCs activates canonical SMAD signaling, reinforcing stemness and promoting immunosuppressive effects (46). IL−6 engages the JAK/STAT3 pathway to drive EMT and self-renewal in CSCs (47). These pathways demonstrate how CSC-derived factors initiate specific signaling cascades that not only sculpt the immune microenvironment but also sustain key CSC phenotypes. Reciprocally, immune cells, especially TAMs, MDSCs, and Tregs, release cytokines such as IL-10, IL-6, and TGF-β, that enhance CSC stemness and chemoresistance through activation of downstream effectors such as STAT3, NF-κB, and Smad signaling pathways (21). Interventions targeting these cytokine/chemokine axes can significantly reverse CSC immune evasion and enhance immunotherapy responses.

Notably, recent studies have revealed that CSC populations exhibit immune evasive adaptations, including downregulation of major histocompatibility complex class I (MHC-I) molecules, expression of immune checkpoint ligands like PD-L1 and B7-H4, and undergoing EMT, all of which specifically diminish recognition and cytotoxicity by CD8^+^ T cells (48). This highlights the complexity of CSC-driven immune evasion and underscores the necessity of incorporating these insights into the design of effective immunotherapies.

### 3.2 Exosome-mediated communication

Exosomes are critical vehicles of intercellular communication between CSCs and immune cells (49–51). CSC-derived exosomes deliver specific RNAs, proteins, and metabolites to immune cells, reprogramming them toward immunosuppressive phenotypes (such as enhancing Treg differentiation, MDSC expansion, and macrophage polarization). Conversely, immune cell-derived exosomes reinforce CSC properties by delivering growth-promoting microRNAs (miR-21, miR-210), signaling proteins, and chemoresistance-associated molecules (37, 38). Given their critical role in the TME, targeting exosomal communication offers promising therapeutic opportunities.

### 3.3 Metabolite exchange mechanisms

Metabolic reprogramming is increasingly recognized as a central mechanism mediating CSC-immune cell crosstalk (52). While both CSCs and non-stem cancer cells are capable of producing immunosuppressive metabolites, CSCs have been shown to exhibit a more sustained and strategic metabolic profile, generating higher levels of metabolites such as lactate, kynurenine, and adenosine. These metabolites inhibit effector immune cell functions and stimulate immunosuppressive cell populations, thereby reinforcing immune evasion and CSC niche maintenance (53–55). Simultaneously, metabolites from immunosuppressive cells (such as TAM-derived metabolites) support CSC growth, survival, and resistance to chemotherapy through metabolic adaptations like enhanced glycolysis and oxidative phosphorylation (56, 57). Therapeutic strategies targeting these metabolic exchanges could disrupt the supportive CSC niche and improve antitumor immunity and chemosensitivity.

## 4 Clinical implications and therapeutic strategies

Given the profound impact of cancer stem cell (CSC) and immune cell interactions on therapy resistance and disease progression, targeting this complex interplay has emerged as a promising therapeutic strategy. A deeper understanding of the molecular mechanisms underlying CSC-immune cell crosstalk provides a valuable basis for developing innovative clinical interventions, either as monotherapies or in combination with existing immunotherapeutic or chemotherapeutic approaches. Here, we outline major clinical implications and discuss ongoing and potential therapeutic strategies targeting these interactions.

Recent evidence has demonstrated that CSC-mediated immune modulation directly contributes to chemoresistance. In breast cancer models, CSCs surviving doxorubicin treatment upregulate TGF−β secretion, which fosters induction of CD4^+^CD25^+^FOXP3^+^ Tregs, promoting an immunosuppressive niche that protects CSCs from both chemotherapeutic and immune-mediated cytotoxicity (58). Moreover, CSCs from various solid tumors enrich PD−L1 expression in response to drug exposure and engage PD−1 on T cells, impairing CD8^+^ T cell function and facilitating survival of therapy-resistant CSC subpopulations (59). These findings highlight that CSC–immune system interactions are not only pivotal for sustaining stemness but are actively involved in the development of chemoresistance, suggesting that effective chemotherapy may require simultaneous disruption of these immune-protective mechanisms.

### 4.1 Preclinical models and translational studies

Preclinical research utilizing animal and patient-derived xenograft (PDX) models has provided critical insights into the mechanisms by which CSC-immune cell interactions promote tumor growth, metastasis, and treatment resistance (60). These effects suggest that CCL2/CSF-1 not only support CSC survival via TAM recruitment but also sustain an immunosuppressive niche that limits T cell activity. Their inhibition leads to CSC depletion and a more immunoreactive microenvironment, thereby enhancing ICI responsiveness. Similarly, studies using CSC-enriched tumor cell populations have shown that blocking exosomal communication between these CSCs and immune cells reduces immune suppression and improves chemotherapeutic efficacy in breast and colon cancer models. Although distinguishing CSC-derived exosomes from those of non-CSCs remains technically challenging, functional studies suggest that CSC-enriched exosomes exert stronger immunosuppressive effects (61, 62). These encouraging preclinical results emphasize the translational potential of targeting CSC-immune cell interactions to sensitize tumors to standard-of-care therapies.

### 4.2 Current clinical trials targeting CSC-immune cell crosstalk

Several clinical trials are currently investigating therapeutic interventions aimed explicitly at disrupting CSC-immune interactions. It is important to recognize that many current clinical trials and therapeutic strategies targeting the immune system or employing epigenetic modulation may influence the broader tumor microenvironment rather than exclusively affecting CSC populations. Consequently, although immune-targeting approaches such as checkpoint inhibitors or epigenetic modulators demonstrate promise, their specificity toward CSCs remains uncertain unless explicitly validated by CSC-specific biomarkers or functional assays. In this review, we include such trials and therapeutic strategies to illustrate potential applications within CSC-driven immune interactions but acknowledge that further studies specifically designed to confirm CSC selectivity and involvement are necessary to clearly distinguish CSC-specific mechanisms from those shared by general cancer cell populations.

**(1) TAM-targeted therapies**

Monoclonal antibodies and small molecule inhibitors targeting macrophage colony-stimulating factor 1 receptor (CSF-1R) have entered clinical trials, aiming to reprogram TAMs toward antitumor phenotypes. For example, the CSF-1R inhibitor Pexidartinib (PLX3397) is being clinically evaluated in combination with immune checkpoint inhibitors (anti-PD-1 or anti-PD-L1) across multiple advanced malignancies (NCT02777710). This strategy is largely specific to TAMs, given the restricted expression of CSF-1R to myeloid cells. CSCs are indirectly affected via disruption of the immunosuppressive niche maintained by TAMs (63). Early-phase results suggest significant modulation of the tumor microenvironment, decreased CSC frequency, and improved immunotherapeutic responses.

**(2) MDSC-targeted strategies**

Therapeutics targeting MDSCs, including CXCR2 inhibitors, have shown potential for reversing immune suppression mediated by CSC-MDSC interactions. A clinical trial of SX-682 (a CXCR1/2 inhibitor) combined with pembrolizumab (an anti-PD-1 immune checkpoint inhibitor) is currently ongoing for advanced solid tumors (NCT03161431) (64). Preliminary outcomes indicate reduced MDSC infiltration and increased cytotoxic T-cell activation, potentially translating into reduced CSC-mediated resistance.

**(3) Treg depletion approaches**

Clinical approaches targeting Tregs, including anti-CCR4 antibodies (such as Mogamulizumab) or IL-2Rα (CD25)-targeted agents, are designed to disrupt the CSC-supportive immune-suppressive niche. Tregs promote CSC maintenance by secreting immunosuppressive cytokines such as transforming growth factor-beta (TGF-β) and interleukin-10 (IL-10), which suppress antitumor immune responses and indirectly support CSC survival and stemness. Therefore, depleting or functionally inhibiting Tregs via CCR4 or CD25 blockade can relieve immunosuppression within the tumor microenvironment, thereby sensitizing CSCs to immune-mediated elimination. Trials evaluating these agents alongside immune checkpoint inhibitors have demonstrated promising antitumor activity, particularly in melanoma and lymphoma (NCT02946671, NCT04189588) (65, 66).

**(4) CSC-targeted vaccines and CAR-T cells**

Cancer vaccines and CAR-T cell therapies specifically targeting CSC surface markers such as CD133, EpCAM, and ALDH are currently under clinical evaluation. For example, CAR-T cells engineered against CSC-specific antigens (CD133 CAR-T) have entered early clinical trials in glioblastoma (NCT03423992) and hepatic carcinoma (NCT02541370), demonstrating favorable safety profiles and preliminary signs of antitumor efficacy (67, 68). Notably, these surface markers are not only used to identify CSCs, but also play functional roles in immune modulation and therapeutic resistance. CD133^+^ glioma stem-like cells have been shown to secrete immunosuppressive cytokines such as TGF-β and promote regulatory T cell recruitment, thereby contributing to an immunosuppressive microenvironment (69). Similarly, CD44^+^ CSCs in breast and colorectal cancers have been linked to upregulation of PD-L1, reduction of CD8^+^ T cell infiltration, and increased chemoresistance (70). These findings support the notion that targeting CSC-associated markers like CD133 and CD44 may directly impact CSC-mediated immune evasion mechanisms. While not all therapeutic approaches discussed in this review exclusively act on CSCs, the vaccine and CAR-T strategies described here represent some of the most promising examples of interventions with demonstrated CSC selectivity and functional impact on the CSC–immune system axis.

### 4.3 Combination therapy strategies to improve clinical outcomes

Given the complexity and redundancy of CSC-mediated immunosuppression, combination therapies represent a more effective approach to comprehensively dismantle these interactions:

**(1) CSC pathway inhibitors and immune checkpoint inhibitors**

Agents targeting canonical CSC-associated signaling pathways (Wnt, Notch, Hedgehog) are now being combined with ICIs. Early-phase trials evaluating the combination of the Wnt inhibitor LGK974 with anti-PD-1 demonstrated improved intratumoral T-cell infiltration and a significant reduction of immunosuppressive myeloid populations, providing strong rationale for further clinical investigation (NCT01351103) (71). Although Wnt signaling is active in various cell types, CSCs exhibit heightened dependency on this pathway for maintaining self-renewal and immune evasion. Thus, Wnt inhibitors such as LGK974 may preferentially impact CSC populations. Nonetheless, off-target effects on immune or stromal cells remain a concern, and ongoing trials are evaluating dosing strategies and biomarkers to enhance specificity.

**(2) Metabolic modulators and immunotherapies**

Metabolic modulators targeting glycolysis, glutamine metabolism, and lipid metabolism, such as CB-839 (glutaminase inhibitor) and CPI-613 (a lipoate analog), are in clinical trials combined with ICIs or chemotherapy (NCT02771626, NCT03399396). These combinations aim to interrupt the metabolic crosstalk that supports CSC immunosuppression, significantly enhancing therapeutic efficacy (72, 73). Emerging studies indicate that CSCs, compared to non-stem cancer cells, exhibit greater reliance on glutamine and lipid metabolism to sustain stemness and immune evasion (74).

**(3) Epigenetic therapy and immunotherapy combinations**

Epigenetic drugs (such as DNA methyltransferase inhibitors or histone deacetylase inhibitors) that target CSC-associated epigenetic modifications are being tested clinically in combination with anti-PD-1 or anti-CTLA-4 therapies. Early data from trials (NCT01928576, NCT03250273) indicate improved antitumor responses potentially through reversing immune suppression mediated by CSC-driven epigenetic reprogramming (75, 76).

### 4.4 Emerging technologies and considerations in clinical implementation

Despite promising early findings, several critical challenges remain for clinical implementation of therapies targeting CSC-immune interactions. Notable concerns include the identification of specific and reliable CSC biomarkers, managing potential toxicities from combined treatments, and overcoming compensatory mechanisms leading to therapy resistance (77). Furthermore, CSC plasticity and heterogeneity can limit the effectiveness of therapies targeting single pathways or markers (78). Future strategies will require integrated biomarker-driven patient selection, real-time monitoring of CSC and immune cell dynamics, and adaptive clinical trial designs to address these complexities effectively.

Novel technologies such as single-cell RNA sequencing, spatial transcriptomics, and multiplexed imaging offer unprecedented insights into CSC-immune cell interactions, guiding more effective therapeutic combinations (79–81). Moreover, leveraging artificial intelligence and machine learning for patient stratification and predictive modeling will further refine therapeutic strategies, enabling personalized interventions tailored to disrupt the specific CSC-immune interaction landscape in individual tumors (82).

In conclusion, targeting the dynamic interplay between CSCs and immune cells holds great promise for overcoming chemoresistance and enhancing the effectiveness of cancer immunotherapies. Although numerous therapeutic strategies, such as immune checkpoint inhibitors and epigenetic modulators, have demonstrated potential clinical efficacy, it is crucial to recognize that these approaches may impact both CSCs and general tumor cell populations within the tumor microenvironment. For instance, clinical trials utilizing PD-1/PD-L1 inhibitors or epigenetic agents like DNA methyltransferase inhibitors have shown encouraging outcomes; however, their specific effectiveness against CSCs requires further validation through CSC-specific biomarkers or assays. Progress in clearly distinguishing CSC-specific mechanisms from general tumor responses will refine therapeutic precision. Continued efforts in biomarker discovery, rigorous clinical evaluation specifically addressing CSC involvement, and technological innovations such as metabolic glycan labeling strategies, exemplified by the recent approach described by Bo et al. (83), will be critical. These integrated advances will pave the way for next-generation anticancer therapies, significantly improving clinical outcomes by effectively targeting CSC-driven therapeutic resistance and immune evasion.

## 5 Challenges and future directions

### 5.1 Challenges in targeting CSC-immune cell crosstalk

Despite significant advances in our understanding of CSC-immune interactions, translating these insights into effective and durable clinical therapies faces several formidable challenges:

**CSC heterogeneity and plasticity:** Cancer stem cells are highly heterogeneous both within and across tumor types. Their phenotypic and functional characteristics vary significantly, influenced by tumor genetic background, microenvironmental cues, and treatment pressures. Furthermore, CSCs exhibit remarkable plasticity, transitioning between stem-like and non-stem-like states in response to environmental changes or therapeutic interventions (84). This dynamic adaptability undermines the effectiveness of therapies targeting static CSC markers or pathways and complicates the identification of universal CSC-specific targets.

**Redundancy of immunosuppressive mechanisms:** CSCs deploy multiple, often redundant mechanisms to establish and maintain an immunosuppressive tumor microenvironment. Even if one pathway is effectively inhibited, alternative compensatory mechanisms may quickly emerge to sustain CSC survival and immune evasion (85). This redundancy limits the efficacy of monotherapies and necessitates the development of rational combination strategies targeting multiple arms of CSC-driven immunosuppression simultaneously.

**Lack of specific and reliable biomarkers:** Identifying and validating specific biomarkers that distinctly define CSCs and their associated immunosuppressive niches remains a major hurdle (86). Notably, several commonly used CSC markers such as CD133, ALDH1, and EpCAM have also been implicated in promoting immune evasion and chemoresistance through modulation of signaling pathways like STAT3 and PI3K/Akt (25). Understanding the dual role of these markers may provide insight into CSC-specific vulnerabilities. Many CSC surface markers (such as CD133, CD44) are also expressed by normal stem cells or other non-malignant cell types, raising concerns regarding on-target off-tumor toxicity. Moreover, dynamic marker expression during disease progression or under therapeutic pressure complicates longitudinal monitoring and patient stratification in clinical settings.

**Toxicity and safety concerns:** Therapeutic strategies that simultaneously target CSCs and modulate immune responses pose risks of systemic immune dysregulation, autoimmunity, or damage to normal tissue stem cells (87). Balancing antitumor efficacy with acceptable toxicity profiles remains a critical challenge, particularly for approaches involving immune checkpoint blockade, Treg depletion, or metabolic reprogramming.

**Inadequate preclinical models:** Current preclinical models often fail to fully recapitulate the complexity of human tumors and the dynamic CSC-immune interactions within the human tumor microenvironment (88). Conventional murine models lack sufficient heterogeneity and immune complexity, which hampers accurate prediction of clinical efficacy and toxicity.

### 5.2 Future directions and promising opportunities

To overcome these challenges and maximize therapeutic potential, several strategic avenues should be prioritized in future research:

**Development of integrated biomarker panels:** Rather than relying on single markers, the integration of multi-parameter biomarker panels combining CSC-specific surface markers, transcriptional profiles, and metabolic signatures may enable more accurate identification and tracking of CSCs *in vivo*. Advances in single-cell RNA sequencing (scRNA-seq), mass cytometry (CyTOF), and spatial transcriptomics offer powerful tools for constructing comprehensive CSC biomarker landscapes, guiding patient stratification and treatment monitoring (89).

**Rational combination therapy design:** Future therapeutic strategies should focus on rationally designed combination regimens that simultaneously target CSC intrinsic pathways (such as Wnt, Notch, Hedgehog) and their immunosuppressive microenvironment components (such as TAMs, MDSCs, Tregs). Preclinical studies suggest that such combinatorial approaches can synergistically enhance antitumor immunity and prevent therapy-induced compensatory mechanisms. Metabolic modulators, by targeting CSC-specific metabolic dependencies (such as glutaminolysis, oxidative phosphorylation), can further disrupt CSC maintenance and reduce the production of immunosuppressive metabolites such as lactate or adenosine, thereby enhancing immune response (90). Clinical trials exploring triple or quadruple combination strategies (such as CSC pathway inhibitors + ICIs + metabolic modulators) are warranted (91).

**Precision immunotherapy targeting CSC niches:** Emerging technologies such as bispecific antibodies, immune engager molecules, and next-generation CAR T-cell designs offer promising avenues for selectively targeting CSCs while sparing normal tissues. Engineering CAR-T cells to recognize dual or conditional antigens associated uniquely with CSCs and their niches may minimize toxicity and improve specificity (92).

**Exploiting metabolic vulnerabilities:** CSC-immune crosstalk is heavily influenced by metabolic reprogramming. Therapeutically targeting metabolic dependencies unique to CSCs and their associated immune cells (such as glutamine metabolism, oxidative phosphorylation, fatty acid metabolism) may offer novel strategies to disrupt the tumor-supportive niche without globally suppressing immune functions (93).

**Real-time monitoring and adaptive therapy:** Dynamic, real-time monitoring of CSC and immune cell interactions during treatment using non-invasive techniques (such as liquid biopsy, circulating exosome profiling, cell-free DNA/RNA sequencing) could enable adaptive therapeutic adjustments to preempt resistance. Integration of artificial intelligence (AI) and machine learning (ML) algorithms for real-time data analysis will further enhance personalized treatment optimization (94).

**Advancement of humanized preclinical models:** Humanized mouse models reconstituted with functional human immune systems and patient-derived tumor tissues provide a more physiologically relevant platform for studying CSC-immune dynamics and evaluating therapeutic strategies. Continued refinement of these models will accelerate preclinical validation and clinical translation (95).

**Metabolic glycan labeling of CSCs:** A promising and innovative approach involves the metabolic labeling of CSCs to enable precise therapeutic targeting. Bo et al. (2023) reported a method using metabolically incorporated azido glycans to selectively tag tumor-initiating cells, allowing for subsequent targeted drug delivery via bioorthogonal click chemistry (83). This glycoengineering platform bypasses the limitations of CSC surface marker heterogeneity and offers a highly selective strategy for identifying and eradicating CSCs. Incorporating such approaches may significantly broaden the translational scope of CSC-directed immunotherapies.

In summary, while significant barriers remain, continued multidisciplinary efforts integrating immunology, cancer biology, systems biology, and advanced biotechnology are steadily advancing the field. By addressing the fundamental challenges and leveraging novel technological innovations, it is conceivable that effective therapeutic strategies targeting CSC-immune crosstalk will emerge, fundamentally reshaping the future landscape of cancer treatment.

## 6 Conclusions

The intricate crosstalk between CSCs and immune cells represents a central mechanism underlying tumor immune evasion, chemoresistance, and relapse. CSC-driven modulation of the tumor microenvironment significantly contributes to immunosuppression and therapeutic resistance, necessitating integrated approaches to overcome CSC-specific immune evasion mechanisms as described above. Targeting these dynamic interactions offers a promising strategy to enhance immunotherapy efficacy and overcome therapeutic resistance. Although substantial challenges remain—including CSC heterogeneity, lack of specific biomarkers, and immune-related toxicities—emerging technologies such as single-cell analysis, spatial omics, and humanized models provide new opportunities for precision targeting. Future therapeutic approaches will likely involve rationally designed combination strategies, integrating CSC-targeted agents with immune modulators and metabolic inhibitors. Real-time monitoring of CSC-immune dynamics and adaptive therapy designs will be essential to maximize clinical benefits. Ultimately, a deeper mechanistic understanding and innovative translational efforts will be key to unlocking the full potential of CSC-immune crosstalk targeting, paving the way for more durable and effective cancer treatments.
